# Advancing integrated care evaluation in shifting contexts: blending implementation research with case study design in project SUSTAIN

**DOI:** 10.1186/s12913-020-05775-5

**Published:** 2020-10-23

**Authors:** Jenny Billings, Simone R. de Bruin, Caroline Baan, Giel Nijpels

**Affiliations:** 1 Centre for Health Service Studies, University of Kent, Canterbury, Kent, CT2 7NF UK; 2 National Institute for Public Health and Environment, Antonie van Leeuwenhoeklaan 9, 3721 Bilthoven, MA Netherlands; 3 VU University Medical Center, De Boelelaan 1117, 1081 Amsterdam, HV Netherlands

**Keywords:** Integrated care evaluation, Implementation research, Case study design, Process and outcome measures

## Abstract

**Background:**

Despite many studies evaluating the effectiveness of integrated care, evidence remains inconsistent. There is increasing commentary pointing out the mismatch between the ability to capture the somewhat ‘illusive’ impact of integrated care initiatives and programmes, and the most appropriate way to do this. Focusing on methodology, this paper describes and critically reviews the experiences of SUSTAIN, a Horizon 2020 funded project (2015–2019) with the purpose of advancing knowledge and understanding of cross-European integrated care evaluation. SUSTAIN sought to improve integrated care initiatives for older people in seven countries, and to maximise the potential for knowledge transfer and application across Europe. The methods approach drew from implementation research, employing the participative Evidence Integration Triangle (EIT) and incorporating a mixed method, multiple embedded case study design. A core set of qualitative and quantitative indicators, alongside context and process data, were created and tested within four key project domains (person-centredness, prevention-orientation, safety and efficiency). The paper critically discusses the overall approach, highlighting the value of the EIT and case study design, and signalling the challenges of data collection with frail older people and stakeholder involvement at the sites, as well as difficulties developing the core set of indicators.

**Conclusions:**

Lessons learned and recommendations for advancing integrated care evaluation are put forward that focus on the status of integrated care as a complex intervention and a process. The use of implementation research methods and case study design are recommended as an additional evaluation approach for researchers to consider, alongside suggested ways of improving methods of data collection with frail populations and cost analysis.

## Background

Despite many studies evaluating the effectiveness of integrated care, evidence remains inconsistent. Increasingly, commentary on the subject of integrated care evaluation is pointing out the mismatch between the ability to capture the impact of integrated care initiatives and programmes, and the selection of the most appropriate methodology to do this. Authors have highlighted a range of evaluation challenges that include the stability and sustainability of initiatives; data collection and the suitability of measures [1, 2]; and a lack of appreciation of the complexity [3]. In addition, the status of integrated care as a ‘process’ must be recognised [4], meaning it is not a ‘fixed’ intervention, but susceptible to constant development and change. These factors all affect the sturdiness of evaluation designs and what constitutes an outcome. This in turn is prompting the need to fit the evaluation design more with how integrated care is implemented in practice and what integrated care is there to achieve and improve [5, 6]. The value of mixed methods studies and suitable frameworks that examine both processes and outcomes has therefore been recognised in this field [2, 7].

Regarding the examination of processes, the wide variation in how integrated care is operationalised calls for evaluations that include a range of qualitative methods, so that important contextual information can be examined to identify what seems to work and why. Regarding outcomes, there is a need to ensure that outcome measures have a good pragmatic fit with the shifting context of integrated care interventions and the population group under study. There is a tendency for example for measures for frail older people and people with multimorbidity to focus on general health outcomes (e.g. health status, physical functioning, quality of life), which may not be appropriate to their fluctuating physical and mental status. Outcomes such as experiences of care, independence and autonomy may be more suited to this vulnerable target group, and these inclusions may be more appropriate to ascertain the link between the integrated care processes and what improvements can be expected for the service user in receipt of care.

Given this impetus, researchers are adopting more ‘real world’ methodologies for the evaluation of complex interventions such as integrated care. While mixed methodologies have been advocated for some time to gain a better appreciation of the ‘grass root’ processes involved in integrated care implementation [8], the emergence of realist approaches drawn from Pawson and Tilley (1997) [9] has become evident [10, 11]. Realist researchers seek to explain the underlying “cause” or mechanisms that generate observed phenomena through the construction of context, mechanism and outcome (CMO) configurations [12]. To support this, academics are developing frameworks such as the COMIC model for the evaluation of integrated care [13]. In addition to realist methods, and continuing with the focus on context, researchers are turning to implementation research. This is described as the scientific study of the processes used in the implementation of an initiative alongside the context within which it is taking place [14]. Its intention is to promote the systematic uptake of research findings and other evidence-based practices into routine practice, and, hence, to improve the quality and effectiveness of health services and care [15].

This broadening of methods appeal is becoming reflected in funding opportunities. The European Union (EU) funded research initiatives (Horizon 2020) are encouraging the adoption of innovative approaches to the evaluation of integrated care to better understand the impact on vulnerable populations with complex needs. One such project is SUSTAIN – Sustainable Tailored Integrated Care for older people in Europe (2015–2019). This paper focuses on the use of innovative approaches within SUSTAIN, and describes the method by which implementation research and case study design were blended for the evaluation of European integrated care initiatives. The aim is to share methodological experiences and lessons learned with the research community, in order to advance understanding of integrated care evaluation in context and add to the international ‘toolbox’ of methodological approaches. It will commence with a brief introduction to SUSTAIN and an overview of the design. This is followed by a critical discussion of the strengths and weaknesses of our approach and concludes with an assessment of the extent to which we advanced understanding of integrated care evaluation. Lessons learned and recommendations for future integrated care evaluations are put forward.

### Overview of the SUSTAIN project

The SUSTAIN project was carried out by thirteen partners from eight European countries: Austria, Belgium, Estonia, Germany, Norway, Spain, the Netherlands, and the United Kingdom. With the exception of Belgium, in all other countries two integrated care initiatives (also referred to as ‘sites’) per country were invited to participate in the SUSTAIN-project, each developing and evaluating two integrated care initiatives (*n* = 14) focusing on older people with complex needs. Sites that were recruited needed to have integrated care initiatives in place, but were motivated to improve and adapt their programmes.

The overall aim of SUSTAIN was to improve integrated care for older people, and to maximise the potential for knowledge transfer and application across Europe [16]. SUSTAIN had four main themes, pre-set by the Horizon 2020 funding programme of person-centredness, prevention-orientation, safety and efficiency in integrated care, which guided the development, implementation and evaluation of the initiatives. The objectives of SUSTAIN were to:

1. Support and evaluate improvements to established integrated care initiatives for older people over 65 living at home with multiple long-term conditions and complex needs; and.
2. Contribute to the adoption and application of these improvements to other health and social care systems, and regions in Europe.

The development and evaluation of SUSTAIN initiatives took place over an 18 month time span, with a 6 month phase of identifying the sites and creating improvement plans, followed by a year long implementation and evaluation period. Ethical approval for the evaluations was obtained through the site-specific governance structures. This paper focuses on the evaluation aspect.

Integrated care comes in many forms, and given that our focus was on developing improvements according to local needs, SUSTAIN has not been exempt from this variability in the selection of initiatives. However, despite the considerable differences in structure and context, two common approaches could be identified within our 14 initiatives;

- Sites that aimed to improve services to older people and/or expand collaboration, communication and coordination with other care and support organisations, while also enhancing knowledge and understanding of each other’s roles and responsibilities;
- Sites that aimed to improve the actual care delivery process to older people, for example providing care in a more person-centred way, or improving case- and discharge management [17].

The challenge for the evaluation design thus became one of developing a robust and consistent approach applicable across all country sites, in the face of several important variabilities. These included differing integrated care configurations and contexts, the site-specific pace of implementation, the throughput of service users and their length of exposure to the intervention, and the enduring problem of data accessibility and comparability [18].

## Methods: the evaluation design of SUSTAIN

### Evidence integration triangle

The overall approach to the project was guided by the Evidence Integration Triangle (EIT). This participatory approach is derived from implementation research and aims to tackle the process of translating research and best-practice evidence to implementation [19].

While evidence on the contribution the EIT is making is still emerging, it is claimed that by focusing on the perspective of stakeholders and the context for application of scientific findings, pragmatic approaches can hasten the integration of research, policy, and practice [20].

**Fig. 1:**
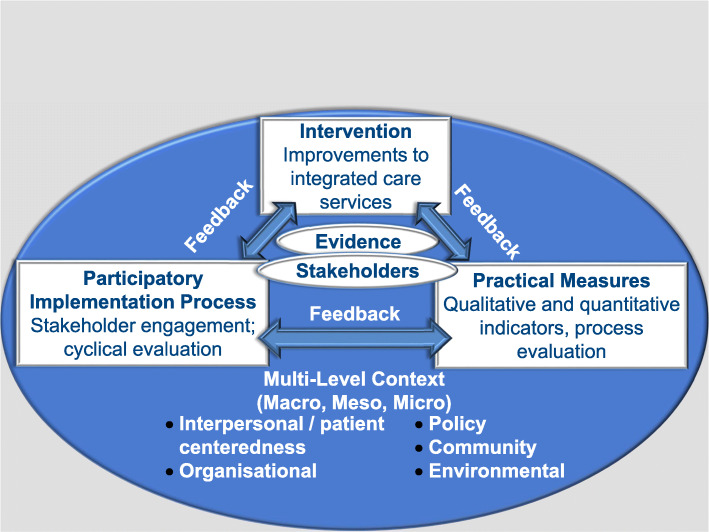
Evidence Integration Triangle [19]

There are three main components to the EIT model, namely the evidence-based *intervention* programme or policy, *participatory implementation processes* with stakeholder involvement, and *practical measures* of progress and outcome (Fig. 1). These three components enable stakeholders to use scientific evidence to encourage development and sharing of new knowledge to inform decision-making. In SUSTAIN, stakeholders were involved by organising steering group meetings. Steering groups consisted of local stakeholders (e.g. managers, health and social care professionals, representatives of older people and carers, commissioners, local policy officers) considered relevant to the integrated care initiative. These steering groups were created to design and implement improvement plans, that is, sets of improvements that apply to local, site-specific priorities.

The high participation levels within this model enabled the research to be relevant and applicable from the onset and ensured that indicators and measures generated to gather evidence remain sensitive of the research and practice environment. Qualitative and quantitative evidence is accumulated and used throughout so that the change process remains dynamic and responsive to improvement.

Context is also pivotal to the EIT. Glasgow [19] describes the multilevel context as the conditions surrounding health problems and intervention opportunities in a particular place with a particular population, and is a key starting point. Context also changes over time, giving a dynamic aspect to the EIT, with context continually informing the other key components. It is clear that this approach was well suited to the fluctuating integrated care environments within which SUSTAIN was taking place.

### Case study design

Within the practical measures aspect of the EIT (the focus of the development of our evaluation tools and approach), we adopted Yin’s [21] case study design. A strength of case study design is its ability to support the analysis of multiple qualitative and quantitative data sources – described as ‘embedded’ - to investigate complex phenomena in their everyday contexts and across different contexts [22]. It was therefore deemed appropriate for examining implementation processes as they unfolded within the EIT cycle in our differing interventions. In addition, it allowed for multiple cases, taking into account the different types of intervention, data source availability and sample size variations across our study sites. As such, with the case study design we aimed to tackle several of the challenges in integrated care evaluation.

Cases are defined by a unit of analysis, common across all sites. With SUSTAIN, our unit of analysis became *‘set of improvements for integrated care initiatives’,* as this was a core objective. In addition, we adopted an explanatory approach to our case study design [21], as we were seeking to develop explanatory models and greater theoretical understanding around two main propositions linked to the four SUSTAIN themes:

1. Integrated care activities will maintain or enhance person-centredness, prevention orientation, safety and efficiency in care delivery;
2. Explanations for succeeding in improving existing integrated care initiatives will be identified.

**Fig. 2:**
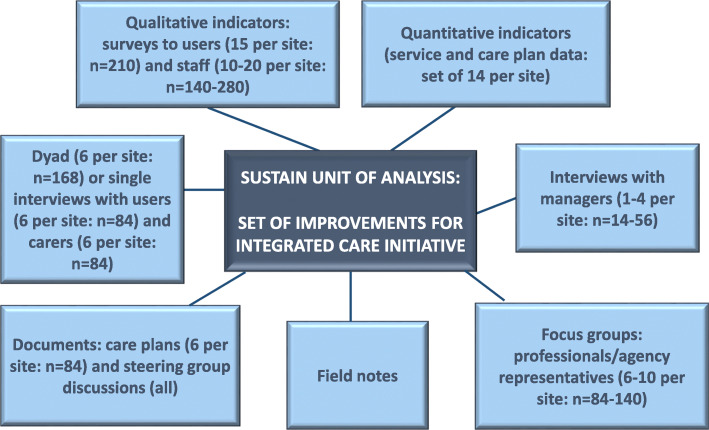
Multiple Embedded Case study design showing data sources and planned samples per site and overall in 14 sites

These propositions were accompanied by a number of analytical questions to support analysis, described in the analysis section. Thus Fig. 2 illustrates our overall approach - multiple embedded case study design that is explanatory in nature**.** In SUSTAIN we differentiated between qualitative and quantitative indicators, a requirement of the Horizon 2020 call. Both produce quantitative data but the former measures attitudes, perceptions and beliefs, and the latter focuses on audit-style data such as hospital admissions [23]. In addition, the figure includes the data sources and the minimum anticipated samples that were seen as achievable, gauged through discussion at partner sites and within the consortium as a whole, taking into consideration the variability previously mentioned such as the differing speed of service user throughput and variable length of the intervention.

### Practical measures

A key feature of the design was to develop and test a core set of indicators that could be used across our partner countries and potentially be transferable to other areas. While Fig. 2 maps out the discreet data sources aligned to case study design, Table 1 unpacks our data sources further, describing distinct data items and data collection tools that were core to the evaluation of our sites in more detail. For clarity and linkage to Fig. 2, qualitative and quantitative indicators are highlighted in colour under the data items column. For data collection tools, instrument selection depended upon the goodness of fit with our objectives and four key themes; availability especially in different languages; validation within our population group; and length. Sites also included some site-specific measures in addition to our core set, that were particular to their interventions, such as audit forms to track new general practice referrals (UK), numbers of GPs, nurses and social workers (Spain), and reasons for not using the integrated care centre for people with dementia (Austria). The selection of our core instruments is elaborated upon and critically reviewed in the discussion.

**Table 1:**
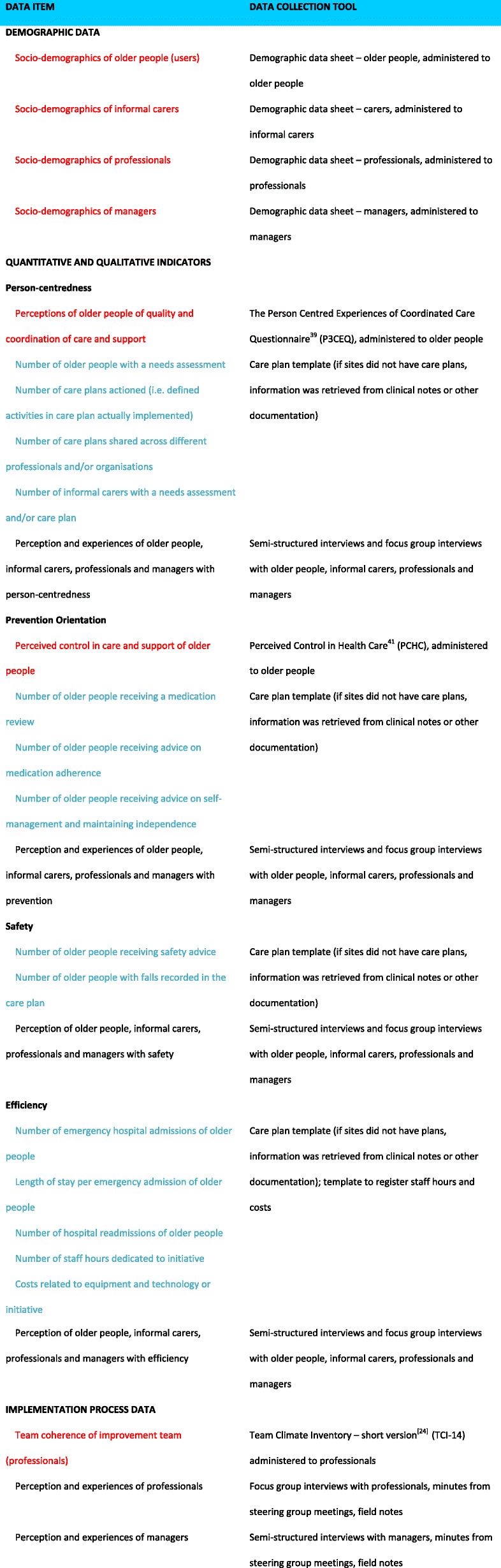
Core Data Items and Data Collection Tools

(Within Data Items, qualitative indicators highlighted in red; quantitative indicators highlighted in blue) [24]

### Data collection

**Fig. 3:**
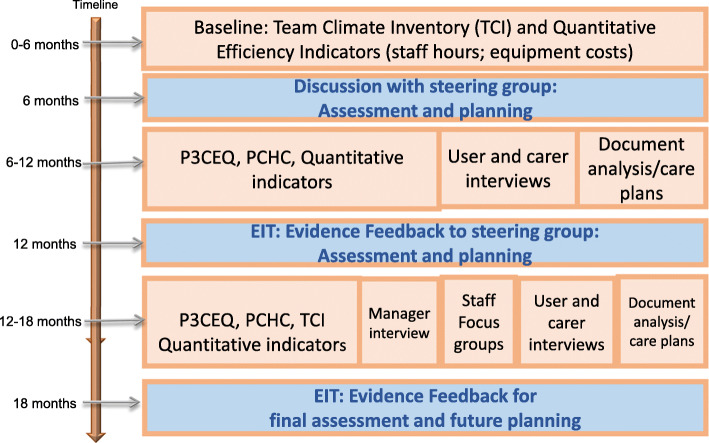
Data collection and Feedback plan

In keeping with the EIT cycles and approach to rapid knowledge transfer, data collection took place over 1 year in two waves following a 6 month development phase where baseline information was collected. Stakeholder reviews of preliminary findings (at the 12 month period) and final findings (at the 18 month period) (Fig. 3) were built in through steering groups to ascertain what seemed to be working well, and where solutions to problems needed to be identified. In order to enable comparison, we used uniform procedures for data collection for all initiatives.

### Analysis

Analysis strategies within multiple case study designs are focused on triangulation of data, purported by Yin [21] to strengthen the construct validity of the research. Each data source became one piece of a jigsaw with each piece contributing to understanding of the whole phenomenon [25]. In SUSTAIN, within each site, data sources were analysed according to their requirements before proceeding to a specific analytical process [26, 27]. Uniform templates for analysis of each data source were generated through a discussion among research partners. All data was entered on a shared anonymised database. Of Yin’s [21] five techniques for analysis, we adopted pattern-matching, seeking rival explanations, linking data to propositions, and explanation building. Exploring rival explanations is an attempt to scan the data to provide an alternate explanation of a phenomenon. To support this, a number of analytical questions were developed to underpin the propositions and our aims, and aid consistency of analytical focus among our evaluation partners:

- What seems to work with what outcomes when making improvements to integrated care?
- What are the explanations for succeeding and improving integrated care initiatives?
- What are the explanations for NOT succeeding and improving integrated care initiatives?
- Are there any factors that can be seen as having an impact on integrated care improvements?
- What factors can be identified that could apply to integrated care improvements across the EU, and be transferable?

Once each site analysis was completed, an overarching cross case synthesis took place. Overall, the evidence created from this type of analysis is considered robust and reliable [28].

## Discussion: addressing the challenges

The discussion will critically review the SUSTAIN design and its appropriateness as an approach to integrated care evaluation. It will firstly discuss the overarching evaluation approach that incorporated the EIT and case study design, follow with a critical reflection on the development of a core set of indicators, and debate the choice of our design in the context of other suitable approaches, namely realistic evaluation.

Reflecting on the EIT, it proved to be highly suitable as a framework for implementation and evaluation for SUSTAIN, in its applicability and use in real-life contexts. A key feature was its practical ability to support a participative environment through the steering group meetings. Here, the framework promoted engagement through its ability to portray a logical and straightforward approach to implementation and evaluation, enabling members to proactively deal with contextual and hence evaluation challenges. It also enabled a level of knowledge exchange and action between the researchers and stakeholders. Other studies are similarly incorporating ‘fit for purpose’ research designs that are placed within the EIT framework. Carrieri et al [29] for example, are undertaking a realist review of interventions to tackle doctors’ mental health, using the EIT to convene a stakeholder group with experts in which research can inform practical decision-making and dissemination of messages. Also Resnik et al [30] are testing the EIT for implementation of interventions to manage behavioural and psychological symptoms associated with dementia, incorporating a pragmatic trial.

Similarly, case study design with its inherent flexibility provided a sound basis for harmonising the disparities between sites and provided a platform to test our core indicators. Yin [21] and Cresswell [31] promote the usefulness of this embedded multi-method design for its ability to add or remove data sources without detriment to the overall analysis. Case study design also gave us a solid data analysis strategy that could accommodate and make comparable and meaningful discrepancies across our partner countries. Case studies have been used in clinical practice and research for a number of decades in complex settings including integrated care [27, 32], as well as within an implementation science approach [33] and in EU studies [34].

In addition, the incorporation of case study design was a significant addition to theory building opportunities (which is somewhat lacking in EIT – see later discussion), going some way towards assembling a deeper theoretical understanding of integrated care. Eisenhardt & Graebner (2007) [35] suggest that theory is emergent, situated in and developed by recognising patterns of relationships among constructs within and across cases. The use of replication logic assisted by pattern matching assists with theory building, in that multiple cases serve as replications, contrasts and extension to the emerging theories. Within SUSTAIN, case study design supported the development of our propositions and consequent explanatory models. Theories embodied within the propositions could be tested and expounded, ultimately leading to theory building, in relation to our central concepts of person-centredness, prevention orientation, safety and efficiency in care delivery, and what seems to ‘work’ in integrated care improvements (see SUSTAIN final report De Bruin et al. 2018) [36].

However our implementation research approach could be described as overly simplistic and lacking clear steps to achieve certain EIT goals, which leaves it open to interpretation. In addition, more guidance is needed regarding how each triangle component relate to each other, as well as how the evidence and stakeholders’ input connect to the triangle individually and as a whole. Importantly, it does not describe sufficiently well how the different context levels should be situated within the triangle and how, within a constantly changing environment, it misses out consideration of sustainability of the intervention. But, the simplicity of the EIT could be described as a strength, in that it is understandable and accessible by participants in the real world, vital in the highly participative stance of the framework. Given the variability within our projects and integrated care interventions generally, the lack of clear process information generated better ‘bottom up’ plans about how the components would work together and relate to the intervention as a whole. Indeed, Glasgow et al. (2012) see other knowledge translation models as too complicated, academic or time consuming for those who wish to use the evidence. In contrast, they purport the EIT to be applicable and usable in a variety of situations, as we found.

With respect to the three key elements within the EIT framework, the practical measures aspect will now be discussed in more detail as it affected the methodological approach. The two other elements, namely evidence-based interventions and the participatory implementation process (including stakeholder involvement), are more concerned with the intervention development and roll out and are reported elsewhere [16, 17, 37]. It is worth, however, mentioning briefly here some experiences with stakeholder involvement as they affected the methodology. In the face of universal health and social care resource constraints, considerable commitment was required for stakeholders not only to develop and implement the improvements with research teams at sites, but also to take part in interviews and assist with obtaining quantitative indicators. Our partnership approach fostered through the EIT approach enabled sustained buy-in to a large extent. However during the course of the implementation plan roll-out, two sites withdrew due to competing priorities and a diversion of resources away from the SUSTAIN initiative. We were able to gather valuable data on the context and reasons for this withdrawal to supplement out analysis. Again, the adoption of case study design overcame these flexes during the data collection period and, overall, helped to create useful and transferable results [31, 38].

Moving now to the practical measures, the extent to which we were able to develop a core set of applicable measures needs consideration. Given the difficulties with integrated care evaluation, we made efforts in our design to select meaningful and pragmatic instruments through a wide literature search, particularly with respect to measuring service user impact. A number of considerations resulted in a contraction of suitable instruments; for example, they had to be applicable to each of the very different integrated care improvements set up within the 14 SUSTAIN sites; they had to be suitable for administration to frail older people; and our central concepts of person-centredness, prevention orientation, safety needed to be reflected in the instruments. Authors have usefully illumined on the evaluation of integrated care and the utility of associated instruments, many of which were considered during the selection process [39, 40]. However, it became clear early on that several existing and validated indicators for frail older people with multimorbidity would be unsuitable. With quality of life measures for example, this was due to the high possibility that relatively short interventions would have little impact; and recommended instruments such as PACIC (Vrijhoef et al. 2009) were not ‘hitting’ all of our considerations sufficiently closely. We therefore narrowed our focus onto an examination of improvements to care and the personal impact of care delivery, which included degrees of person-centredness, experiences of co-ordination, and perceived control and independence.

With this in mind and after much deliberation within the SUSTAIN consortium, we selected the P3CEQ [41, 42], and the PCHC [43], the latter validated for our population group. At the time of selection, the P3CEQ was relatively new but seemed suitable for administration across all sites and intervention types. We did experience, however, some repetition between these two questionnaires, and in some sites there were significant problems with recruitment and fatigue of older people. In response to this, the PCHC was withdrawn, as the P3CEQ seemed more tuned to the SUSTAIN themes and also included items on control and independence in health and social care. Case study design accommodated this adaptation. The data collection and analysis relating to the P3CEQ was not without its challenges during the course of SUSTAIN however. We found it needed essential preconditions (eg. face-to-face administration, collections of reasons for non-response) and administration and coding guidelines (eg where informal carers support service users to answer questions) [44]. We conclude through our experiences, that establishing a solid and standard cross-country measure of older service user experiences for integrated care still remains fraught with complexity and somewhat elusive.

Obstacles were more apparent with obtaining quantitative indicators due to the availability, accessibility and reliability of appropriate health and social care data across partner countries. This is due for example to differences in what and how data is collected, variations in the geographical representation of data, and the general lack of social care data, and these problems are persistent. For example, across Europe, data is scattered across systems, is not interoperable, and there are privacy concerns and technical challenges that block effective data recording and sharing at local, national and European levels [18]. In addressing this somewhat ‘hostile’ environment, we co-created a core list with professionals and managers at the sites of what could be obtained from either routine service level data, clinical notes, care plans or other sources (see Efficiency data in Table 1 for indicators that were deemed common across sites).

Collecting directly from clinical data and care plans had the potential to be a rich source of data [45] and could overcome the problem of aggregated measures, such as hospital admission data, and their sensitivity to projects where the population group is small and widely dispersed. Similarly, with the cost data, very few sites were able to extract specific costs related directly to the improvement interventions, but an estimate of staff hours was deemed possible, to give some indication of resource use. However using both clinical notes, care plans and staff hours were dependent upon the accurate recording of these events by busy practitioners and managers, which could not be assured, an aspect also acknowledged by Jefferies et al [46] With clinical notes, this recording was variable and unless prompted, did not always yield the information required. Care plans were not always completed or available; other researchers have had similar experiences and list causes as staff time pressures, poor document construction and communication difficulties with service users, recognised in other studies [7]. With staff hours, although diaries/templates were made available at sites, staff worked across initiatives and were not always able to separate and accurately record specific hours dedicated to the improvement initiatives. So, in most cases this was estimated, and thus the ability to give a sound cost analysis was greatly reduced.

With this last point, difficulties with the measurement of cost in integrated care is the subject of much debate within the literature. Lack of standardised outcomes and continuous changes in care delivery, for example, render the employment of traditional economic models unusable [47]. While SUSTAIN was keen to avoid health economic methods that have a poor fit with the nature of integrated care, it was clear that our more pragmatic approach was also not optimal, and the search for a more reliable and attributable method should continue.

Any deficits within quantitative data were however compensated by the richness of our qualitative data sources. As well as service user and carer interviews, we obtained professional, managerial and other stakeholder viewpoints, alongside documentary evidence from care plans (where available), steering group meetings and field notes. These perspectives provided valuable insights into personal impacts of the intervention, contextual influences and more nuanced information about if, how and why improvements made a difference (see De Bruin et al. [36]).

Having reviewed the relative mertis of the EIT framework, the discussion moves on to a critique of case study design. One of the most commonly cited disadvantages of case studies is that findings can lack generalisability and scientific credibility because replication is difficult [37]. However, external validity can be stronger in multiple case study designs, which was the choice in SUSTAIN, and can be weak in more highly ranked randomised control trials. Such weaknesses in RCT design have been exposed in a number of systematic reviews and secondary analyses [48].

In practical terms, there are further difficulties that researchers can encounter. For example, there can be a tendency to become overwhelmed with data and the process can be very time consuming, particularly with regard to developing and blending thematic statements from the analysed data sources. This occurs particularly when propositions are lacking and there has been no attempt to link the data collection with the aims of the study in a focused way, or create some boundaries to data collection [26]. In SUSTAIN, we established clear objectives and propositions, protocols for every aspect of data collection and management, analytical templates for ensuring consistency with data analysis, and a shared quality-controlled database. Difficulties still arose however, so to supplement this and optimise uniformity of our evaluation approach, we arranged regular one-to-one progress and ‘trouble-shooting’ calls with research teams and devoted space at six monthly consortium meetings to deal with methods issues.

The discussion now moves finally to a consideration of why we selected implementation research over other methods such as realist evaluation. For SUSTAIN, the importance of gaining a consistent and understandable method across different institutions and contexts, as well as involving stakeholders not wholly conversant with research, was paramount. While our approach was not fault-free, realist methods also has its challenges regarding its complexity. For example, Greenhalgh et al. (2009) [10] noted that a set of more or less well-defined ‘mechanisms of change’ in reality can prove difficult to nail, and the process of developing CMO configurations is an interpretive task, achieved through much negotiation and dispute. In addition, the authors add that while realist evaluation can draw useful lessons about how particular preconditions make certain outcomes more likely, it cannot produce a simple recipe for success. Given that this latter aspect was a significant factor for our aim of promoting good knowledge transfer, the applicability of realist approaches to our design was limited, with implementation research seemingly more suitable.

Nevertheless, similarities are evident between these different evaluation approaches. While realist uses the development of CMO configurations, implementation research also investigates equally important factors affecting implementation (geographical, cultural beliefs, poverty), the processes of implementation themselves (multi-disciplinary working, local resource distribution) and the end product or outcome of the implementation [14]. Implementation research does not however link the components so strongly, circumnavigating the lengthy interpretation tendency of realist approaches. Nor does it, particularly in the case of EIT, lend itself to so readily to theory generation, unlike realist approaches. Hence combining the EIT framework with case study design as we did in SUSTAIN offered stronger opportunities for theory testing and development, as previously outlined.

## Conclusions: lessons learned and recommendations

Overall, in the strive to seek out the answers to ‘what works’ in integrated care provision, SUSTAIN has enabled the identification of different ways to advance integrated care evaluation locally, nationally and across Europe, that fundamentally recognises its status as a complex intervention, and as a process. Operating within this conceptual and theoretical understanding, we were able to apply pre-emptive consideration to the challenges in the evaluation design, obtaining a good pragmatic fit with the objectives of evaluating improvements. It is clear that difficulties with health data continue, which impacted on our ability to provide a robust transferable set of core indicators, highlighting the continuing challenges. However, instruments within this set still are anticipated to be of value and more meaningful to what integrated care should aim to achieve. Integrated care evaluation continues to challenge, and our approach in SUSTAIN was not without its own challenges. However out intention with this paper is to support researchers by adding to the international methodological repertoire of evaluation approaches that encourage a goodness of contextual fit.

The following are key lessons learned and recommendations:

1. Without doubt, we would advocate a participatory approach to evaluation designs and one set within implementation research. This recognises the dynamic nature of integrated care implementation and keeps pace with its ebbs and flows, thereby strengthening the evaluation approach and potential for knowledge transfer.
2. Case study design also proved to be highly useful and adaptable to the changes in evaluation requirements, variations between sites, and is pertinent to cross-European comparative research.
3. With respect to the target group of older people, there is a clear need to employ more innovative data collection techniques that step aside from traditional survey and interview approaches, towards methods that are interactive, engaging and experiential and take account of ageing. Talking Mats, a tested and validated vehicle to support older people to communicate about things that matter to them, is gathering momentum as a research tool [49] and may be a way forward.
4. Further research is needed to better understand and measure the relationship between resource and cost changes and integrated care. In keeping with growing opinion, the focus must move away from traditional health economic models towards a more realistic and pragmatic perspective of what can be measured. Rephrasing of cost objectives towards investigating a ‘better use of resources’ within the integrated care environment may be a start.

## Data Availability

Researchers can apply for data by submitting a proposal to g.nijpels@vumc.nl. After agreement of the proposal analysis by the SUSTAIN steering committee, and after ethics approval and a data transfer agreement, collaborative researchers can receive data for a specific research question. Fees will be dependent upon the amount of work needed for data extraction.

## Abbreviations

EIT: Evidence Integration Triangle
EU: European Union
P3CEQ: Person-centred experiences of co-ordinated care questionnaire
PCHC: Perceived control of health care
TCH-14: Team climate inventory short version

## References

[1] Billings J, Leichsenring K, Billings J, Nies H (2013). Improving the evidence base. Long-term care in Europe: improving policy and practice.

[2] Nuño Solinís R, Stein KV (2016). Measuring integrated care – the quest for disentangling a Gordian knot. Int J Integr Care.

[3] Tsasis P, Evans JM, Owen S. Reframing the challenges to integrated care: a complex-adaptive systems perspective. International Journal of Integrated Care. 2012;12(5). 10.5334/ijic.843. 10.5334/ijic.843 PMC3601537 23593051

[4] Shaw S, Rosen R, Rumbold B. What is integrated care? Nuffield trust; 2011. https://www.nuffieldtrust.org.uk/files/2017-01/what-is-integrated-care-report-web-final.pdf Accessed 15th Nov 2018.

[5] De Bruin SR, Versnel N, Lemmens LC, Molema CCM, Schellevis FG, Nijpels G (2012). Comprehensive care programs for patients with multiple chronic conditions: a systematic literature review. Health Policy.

[6] Billings JR, Leichsenring K. Methodological development of the interactive INTERLINKS Framework for Long Term Care. International Journal of Integrated Care. 2014;14(2). 10.5334/ijic.1173. 10.5334/ijic.1173 PMC4109401 25120413

[7] Curry N, Harris M, Gunn L, Pappas Y, Blunt I, Soljak M, et al. Integrated care pilot in north West London: a mixed method evaluation. *Int J Integr Care*. 2013; 13 25th July https://www.ijic.org/articles/10.5334/ijic.1149/ Accessed 15th Nov 2018. 10.5334/ijic.1149 PMC3807631 24167455

[8] Billings J. The INTERLINKS Framework for Long-Term Care of Older People in Europe. Journal of Integrated Care. 2013;21(3):126–38. 10.1108/JICA-02-2013-0007.

[9] Pawson R, Tilley N (1997). Realistic evaluation.

[10] Greenhalgh T, Humphrey C, Hughes J, Macfarlane F, Butler C, Pawson R (2009). How do you modernise a health service? A realist evaluation of whole-scale transformation in London. Milbank Q.

[11] Middleton L, Rea H, Pledger M, Cumming J (2019). A realist evaluation of local networks designed to achieve more integrated care. Int J Integr Care.

[12] Jagosh J, Bush PL, Salsberg J (2015). A realist evaluation of community-based participatory research: partnership synergy, trust building and related ripple effects. BMC Public Health.

[13] Busetto L, Luijkx K, Vrijhoef B (2017). Development of the COMIC model for the comprehensive evaluation of integrated care interventions. Int J Integr Care.

[14] Peters DH, Adam T, Alonge O, Akua Agyepong I, Tran N (2013). Implementation research: what it is and how to do it. BMJ.

[15] Eccles MP, Mittman BS (2006). Welcome to *Implementation Science*. Implement Sci.

[16] De Bruin SR, Stoop A, Billings J, Leichsenring K, Ruppe G, Tram N (2018). The SUSTAIN project: a European study on improving integrated Care for Older People Living at home. Int J Integr Care.

[17] Stoop A, De Bruin SR, Wistow G, Billings J, Ruppe G, Leichsenring K (2019). Exploring improvement plans of fourteen European integrated care sites for older people with complex needs. Health Policy.

[18] Politico (2017). Europe chases promise of big health data.

[19] Glasgow RE, Green LW, Taylor MV, Stange KC (2012). An evidence integration triangle for aligning science with policy and practice. Am J Prev Med.

[20] Glasgow R (2013). What does it mean to be pragmatic? Pragmatic methods, measures and models to facilitate research translation. Health Educ Behav.

[21] Yin RK (2009). Case study research: design and methods.

[22] Swanborn P (2010). Case study research: what, why and how?.

[23] Church C, Rogers MM (2006). Indicators. Chapter 4 in *Designing for Results: Integrating Monitoring and Evaluation in Conflict Transformation Programs*.

[24] Kivimäki M, Elovainio M (1999). A short version of the team climate inventory: development and psychometric properties. J Occup Organ Psychol.

[25] Hancock DR, Algozzine B (2006). Doing case study research: a practical guide for beginning researchers.

[26] Billings J (2004). Towards rigour in qualitative research across European partnerships. Eur J Ageing.

[27] Donnelly C, Brenchley C, Crawford C, Letts L (2013). The integration of occupational therapy into primary care: a multiple case study design. BMC Fam Pract.

[28] Baxter, Jacks (2008). Qualitative case study methodology: study design and implementation for novice researchers. Qual Rep.

[29] Carrieri D, Briscoe S, Jackson M, et al. Care under pressure’: a realist review of interventions to tackle doctors’ mental ill-health and its impacts on the clinical workforce and patient care. BMJ Open. 2018:e021273. 10.1136/bmjopen-2017-021273 Accessed 14th Oct 2018. 10.1136/bmjopen-2017-021273 PMC5829880 29420234

[30] Kolanowski A, Van Haitsma K, Galik E, Boltz M, Ellis J, Behrens L (2018). Testing the evidence integration triangle for implementation of interventions to manage behavioral and psychological symptoms associated with dementia: protocol for a pragmatic trial. Res Nurs Health.

[31] Creswell J (2009). Research design; qualitative and quantitative and mixed methods approaches.

[32] Bergen A, While A (2000). A case for case studies: exploring the use of case study design in community nursing research. J Adv Nurs.

[33] Chambers A, Mustard CA, Breslin C, Holness L, Nichol K. Evaluating the implementation of health and safety innovations under regulatory context: a collective case study of Ontario’s safer needle regulation. Implement Sci. 2013:8–9 www.implementationscience.com/contents/8/1/9 Accessed 12th Dec 2018. 10.1186/1748-5908-8-9 PMC3556097 23339295

[34] Eerden M, Csikos A, Busa C, Hughes S, Radbruch L (2014). Experiences of patients, family and professional caregivers with integrated palliative care in Europe: protocol for an international, multicentre, prospective, mixed method study. BMC Palliat Care.

[35] Eisenhardt KM, Graebner ME (2007). Theory building from cases: opportunities and challenges. Acad Manage J.

[36] De Bruin SR, Stoop A, Baan CA, Billings JR, Nijpels G, on behalf of the SUSTAIN consortium (2018). Sustainable tailored integrated care for older people in Europe (SUSTAIN-project).

[37] MacInnes J, Baldwin J, Billings J (2020). The over 75 service: continuity of integrated Care for Older People in a United Kingdom primary care setting. Int J Integr Care.

[38] Raeburn T, Schmied V, Hunderford C, Cleary M (2015). The contribution of case study design to supporting research on clubhouse psychosocial rehabilitation. BMC Res Notes.

[39] Vrijhoef HJM, Berbee R, Wagner EH, Steuten LMG (2009). Quality of integrated chronic care measured by patient survey: identification, selection and application of most appropriate instruments. Health Expect.

[40] Bautista MAC, Nurjono M, Lim YW, Dessers E, Vrijhoef HJ (2016). Instruments measuring integrated care: a systematic review of measurement properties. Milbank Q.

[41] Lloyd H, Fosh B, Whalley B, Byng R, Close J (2019). Validation of the person-centred coordinated care experience questionnaire (P3CEQ). Int J Qual Health Care.

[42] Sugavanam T, Fosh B, Close J, Phil D, Byng R, Horrell J (2018). Co-designing a measure of person-centred coordinated care to capture the experience of the patient. J Patient Experience.

[43] Claassens L, Terwee CB, Deeg DJ, Broese van Groenou MI, Widdershoven GA, Huisman M (2016). Development and validation of a questionnaire assessing the perceived control in health care among older adults with care needs in the Netherlands. Qual Life Res.

[44] Reynolds J, Gadsby E, Rijken M, Stoop A, Espallargues M, Lloyd H, et al. Measuring older peoples’ experiences of person-centred coordinated care: the SUSTAIN experience applying a patient reported experience measureIn press. Int J Integr Care. 2020. in press. 10.5334/ijic.5504 PMC8284500 34305488

[45] Andrews A, St Aubyn B (2015). If it’s not written down it didn’t happen. J Commun Nurs.

[46] Jefferies D, Johnson M, Griffiths R (2010). A meta-study of the essentials of quality nursing documentation. Int J Nurs Pract.

[47] Evers SMMA, Paulus ATG. Health economics and integrated care: a growing and challenging relationship. Int J Integr Care. 2015;15 17 June 2015, URN:NBN:NL:UI:10-1-114829 https://ijic.ubiquitypress.com/articles/10.5334/ijic.2201/ Accessed 14th Nov 2018. 10.5334/ijic.2201 PMC4491323 26150762

[48] Hunt G, Siegfried N, Morley K, Sitharthan T, Cleary M (2013). Psychosocial interventions for people with both severe mental illness and substance misuse (review). Cochrane Database Syst Rev.

[49] Murphy J, Oliver TM (2013). The use of talking Mats to support people with dementia and their carers to make decisions together. Health Soc Care Community.

